# Erosion–Corrosion of 30°, 60°, and 90° Carbon Steel Elbows in a Multiphase Flow Containing Sand Particles

**DOI:** 10.3390/ma12233898

**Published:** 2019-11-26

**Authors:** Rehan Khan, Hamdan H. Ya, William Pao, Armaghan Khan

**Affiliations:** 1 Mechanical Engineering Department, Universiti Teknologi PETRONAS, Bandar Seri Iskandar, Perak 32610, Malaysia; william.pao@utp.edu.my; 2 Department of Mechanical Engineering, McGill University, Macdonald Engineering Building, 817 Sherbrooke Street West Montreal, Montreal, QC H3A 0C3, Canada; armaghan.khan@mail.mcgill.ca

**Keywords:** erosion–corrosion, slug flow, multilayer paint modeling, surface roughness, microscopic surface imaging

## Abstract

Erosion–corrosion in flow changing devices as a result of sand transportation is a serious concern in the hydrocarbon and mineral processing industry. In this work, the flow accelerated erosion–corrosion mechanism of 90°, 60°, and 30° long radius horizontal–horizontal (H–H) carbon steel elbows with an inner diameter of 50.8 mm were investigated in an experimental closed-flow loop. For these geometrical configurations, erosion–corrosion was elucidated for erosive slug flow regimes and the extent of material degradation is reported in detail. Qualitative techniques such as multilayer paint modeling and microscopic surface imaging were used to scrutinize the flow accelerated erosion–corrosion mechanism. The 3D roughness characterization of the surface indicates that maximum roughness appears in downstream adjacent to the outlet of the 90° elbow. Microscopic surface imaging of eroded elbow surfaces disseminates the presence of corrosion pits on the exit regions of the 90° and 60° elbows, but erosion scars were formed on the entry regions of the 30° elbow. Surface characterization and mass loss results indicated that changing the elbow geometrical configuration from a small angle to wide angle significantly changed the mechanical wear mechanism of the tested elbows. Moreover, the maximum erosive location was identified at the top of the horizontally-oriented elbow for slug flow.

## 1. Introduction

Carbon steels are a widely used engineering material in hydrocarbon production pipelines. In defiance of their excellent mechanical and thermal properties, the presence of entrained sand in accelerated carrier fluids enhances erosion-induced damage, which depresses the integrity of the transportation pipelines under field operating conditions. Much work [1,2,3,4,5,6,7,8,9,10,11,12,13] has been directed to understanding the failure mode of ductile material and proposing models and mechanisms to decouple the relationship between different physical parameters and erosion rates under specific experimental conditions. In solid particle erosion–corrosion, an increase of impact velocity results in catastrophic damage and greater mass transfer and escalates the erosion–corrosion rate [12,14,15]. Aminul et al. [16] found that in slurry erosion–corrosion, the surface roughness of the pipeline increases the corrosion rate owing to the higher surface area of the worn surfaces. Telfer et al. [17] observed that increasing the particle concentration strongly influences the erosion–corrosion rate of ductile materials.

Erosion in pipelines with gas, liquid, and solid phases is complex in nature and strained by phase interaction, hydrodynamics, disperse phase characteristics, pipe configurations, and material characteristics. Slug flow regime occurrence under turbulent conditions is commonly observed in hydrocarbon production pipelines. This flow regime consisted of the high-flow rate gas phase above the pipeline and the liquid film that travels below the gas phase, and the highly turbulent slug front can accelerate erosion-induced damage in pipelines [18,19].

In the last decade, many researchers tried to understand sand-multiphase mixture erosion, but no significant understanding of erosion mechanism has been developed, especially in the erosive slug flow pattern ascribed to the complexity of the carrier phase and dispersed phase interactions.

Kesana et al. [20] carried out the erosion study to extract the relationship of particle size and carrier fluid viscosity in a standard elbow 90° horizontal for pseudo slug/annular flows and concluded that the sand diameter has a direct influence on the pipeline erosion. Vieira et al. [21] performed several experimental tests on 90° elbow erosion in the sand-multiphase mixture. He reported that the sand erosion is strongly reliant on the elbow orientation. Parsi et al. [22] recently performed a CFD study to understand solid particle erosion for pseudo-slug flow pattern in the horizontal 90° elbow. The results showed that the presence of liquid film affects sand transport velocity and depleted particle momentum.

Sand particles entertained in slug flow poses an erosion threat owing to liquid–sand slug impingement on the pipe and flow devices’ walls with the redirected flow of more than one carrier phase, and leads to equipment damage due to flow accelerated erosion–corrosion and overstressing of flow changing devices (i.e., elbow, tee junction) [19,23]. Considering the complexity of the multiphase sand transport in the specific domain of predicting erosion for gas–liquid–solid flows [20,21,24,25,26], there is a need to acquire useful experimental data to understand the erosion–corrosion mechanism of elbow configurations. In particular, the extent of erosion-induced damage from sand entrained in air–water slug flow is not fully understood. Moreover, the influence of the elbow angle on the mechanism of material degradation for the slug flow regime is still not completely clear.

To optimize the hydrocarbon production process, it is important for engineers and designers to have tribological information about the erosion–corrosion process. This specific study of erosion–corrosion due to solid sand particles should be able to provide the appropriate information on the elbows’ erosion–corrosion degradation mechanism for operating conditions encountered in oil and gas fields.

In this work, the erosion–corrosion performance of long radius 90°, 60°, and 30° carbon steel elbows with 50.8 mm inner diameters is evaluated by close flow loop experiments for slug flow. The carrier phases are water and air, and the erodent is fine sand of size 50 ± 2 µm with 2% (wt/wt) particle concentration. In addition, the liquid phase superficial velocity of 0.5 m/s, and gas-phase superficial velocity of 2.5 m/s, are set in all cases evaluated. The direct metal loss of the upper half and bottom half sections of the elbows are measured with precision balance. The surface characterization of the internal surface of the studied elbows was extracted with 3D non-contact profiling and microscopic imaging techniques. This study aims to determine the erosion–corrosion mechanism in three different elbow configurations by employing multilayer paint modeling, 3D surface roughness, confocal microscopic imaging, SEM/EDX, and weight loss measurements. 

## 2. Experimental Methods and Materials

**Figure 1:**
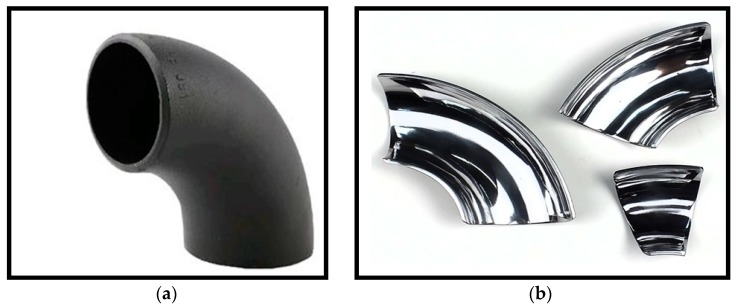
Carbon steel elbow specimen. (**a**) As received, and (**b**) fine polished axially cut sections.

**Figure 2:**
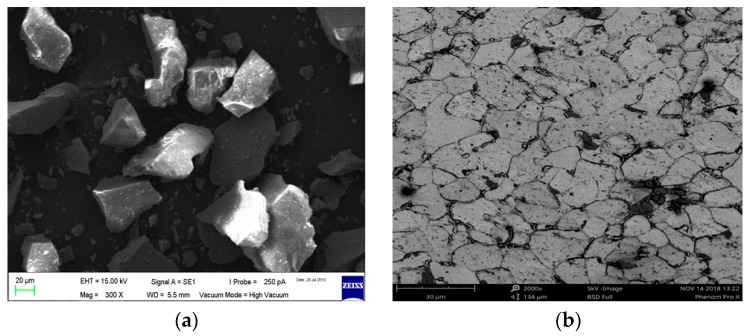
Microstructures of (**a**) silica sand and (**b**) carbon steel.

The target carbon steel elbow configuration used for the erosion tests was axially cut using an EDM wire cut machine in two sections, the bottom half (BH) and upper half (UH), as outlined in Figure 1. The test elbows’ inner walls were ground using 400, 600, 800, and 1200 grit size of the sandpapers and then polished using 1 μm crystalline diamond suspension, degreased in ethanol, dried, and placed in desiccators to avoid exposure of the sample to moisture before the tests. The material loss was measured using a METTLER TOLEDO precision balance and the test elbow chemical composition listed in Table 1 was extracted by the optical emission spectroscopy method. Carbon steel was used as the test material and fine silica sand was used as a dispersed phase in all erosion experiments. The sand size distribution was quantified by means of a laser scattering particle size distribution analyzer. The result gives an average erodent size of 50 ± 2 µm. A microstructure of the silica sand and test material is presented in Figure 2. Surface characterization was done by a Sensofar S lynx microscope in confocal mode and the surface parameter was computed at the inlet, middle, and outlet sections of all tested elbows. Surface profiling was conducted to obtain 3D topographies of the worn surface and to determine the surface roughness of the 90°, 60°, and 30° elbows. Specimens of 10 mm × 10 mm in size were cut using EDM from the entry, middle, and exit regions of elbows after the erosion–corrosion test for the surface characterization. The most significant surface roughness parameters are the arithmetic mean surface parameter and maximum pit depth. The arithmetic mean height (S_a_) is the mean surface roughness of the worn surface, while the maximum pit depth (S_v_) gives information about the pit depth distribution. In order to extract roughness parameters of the assessed 3D surface topography, waviness components are suppressed and filtered out using areal Gaussian filter (ISO 16610-61).

### 2.1. Multiphase Flow Loop and Medium

**Figure 3:**
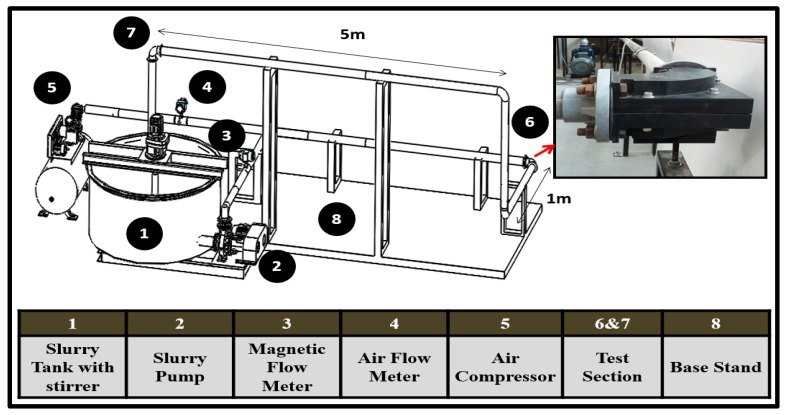
The experimental setup used for the present investigation.

The multiphase flow loop experimental facility at Universiti Teknologi PETRONAS is locally fabricated and configured (Figure 3), where different geometric elbow configurations can be tested for gas–liquid–solid slug flow conditions. The flow loop consists of a transparent pipe section of 5 m in length to visualize flow pattern inside pipelines, and the 50.8 mm elbows test section (Figure 3) is mounted in a horizontal–horizontal orientation, where the elbow BH and UH sections are placed, and erosion-induced damage is studied using the multilayer paint modeling and material loss methods. The carrier phases are water and air for the present study, while the dispersed phase is sub-angular fine sand. The chemical compositions of silica sand are listed in Table 2. Although the experimental setup has two elbows mounted in a horizontal–horizontal and horizontal–vertical orientation, in this study, only the horizontal–horizontal orientation will be addressed. The experimental data point for slug flow is selected based on Madhane et al.’s [27] flow pattern map.

### 2.2. Test Section

**Figure 4:**
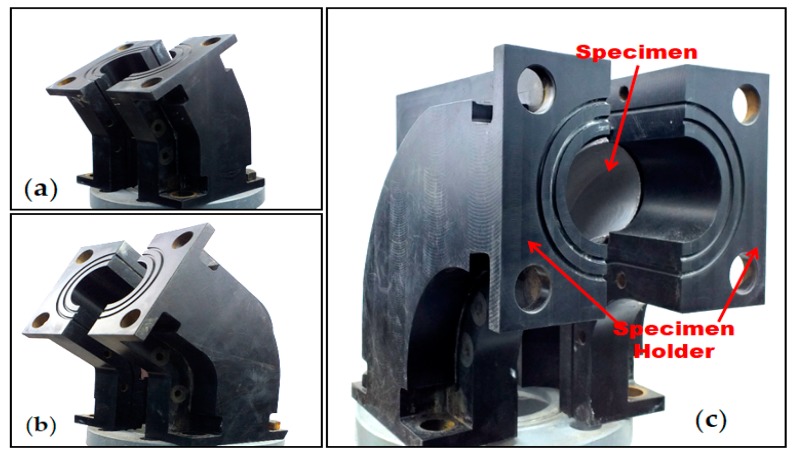
Section of (**a**) 30° (**b**) 60°, and (**c**) 90° elbows.

The test specimen holders of the experimental setup (Figure 4a–c) were fabricated with thermoplastic material with the cavity of the half elbow pipe configuration machined using a CNC machining unit to place the test elbow inside the test section. To identify the erosion zones and the erosion severity inside the elbow configuration, the elbow pipe is cut axially in two sections, the bottom half (BH) and upper half (UH), before the test and painted with two layers of enamel paint on the internal surface. From the paint removal of the first layer, it is possible to identify the maximum erosive location inside the elbow pipe. Figure 4c shows an elbow sample mounted inside the test section, fabricated with two thermoplastic blocks with the cavity of elbow inside the blocks. For each experiment, the block was bolted together after placement of the sample to make an elbow pipe, and three samples are tested for repeatability and accuracy of the test. Then, a visual inspection is performed to identify the removed paint locations, and those paint erosion patterns give an approximation of the location of the maximum particle collision. The maximum erosion observed utilizing paint removal patterns towards the end of the study is compared to that utilizing the paint removal patterns in different elbow angular configurations to understand erosion mechanism. After identification of maximum erosion zones, the test was repeated with the polished specimens for quantification of the erosion–corrosion rate. All multilayer paint tests were repeated multiple times to ensure accuracy. However, in the erosion–corrosion quantification, the uncertainty originates from the non-uniform surface finish of the polished specimen’s internal surface due to curvature, which influences the impact conditions and mechanistic behavior of the surface. The eroded weight was measured to quantify the erosion–corrosion rate in kg/s·m^2^ as follows:

(1) $$\mathrm{Erosion}\;-\;\mathrm{Corrision}\;\mathrm{Rate}\;=\;\frac{\left(\mathrm{Intial}\;\mathrm{Weight}\;-\;\mathrm{Final}\;\mathrm{Weight}\right)}{\mathrm{Impact}\;\mathrm{Area}\;\times \;\mathrm{Test}\;\mathrm{Time}}$$

## 3. Results and Discussion

### 3.1. Multilayer Paint Modeling (MPM) to Determine Erosion Patterns

Multilayer paint modeling (MPM) is an inexpensive qualitative visual tool for accurately mapping the erosion location in significantly less time, using the flow loop at high transportation velocities [28,29,30]. The MPM technique is a qualitative tool and cannot yield any quantitative data related to the erosion–corrosion rate of the pipe wall. In MPM, multilayer paints (15–30 μm thick) with a red layer followed by a silver layer were applied to the inner elbow surface before the test. Before the test, the pipe inner surface degreased in ethanol to eliminate the residual contaminant, and each paint layer was dried before the application of imminent layers. After each test, the elbow sample was unmounted from the test section for visualization of the paint removal location on the inner elbow wall. Nevertheless, the MPM technique will provide a qualitative approximation of the erosion distribution on the pipe wall.

**Figure 5:**
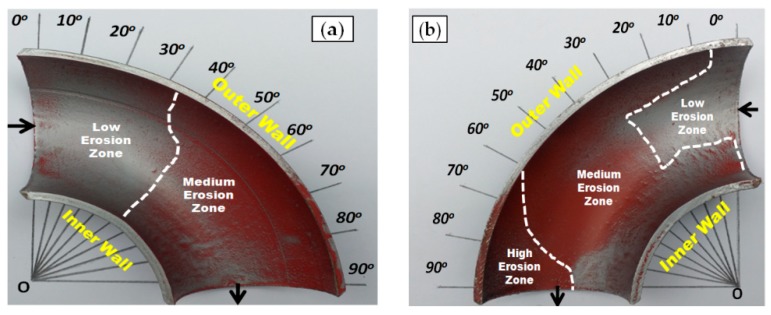
Paint erosion pattern for slug flow with Vs_L_ = 0.5 m/s, Vs_G_ = 2.5 m/s in a 90° horizontal–horizontal elbow section. (**a**) Bottom half; (**b**) upper half.

**Figure 6:**
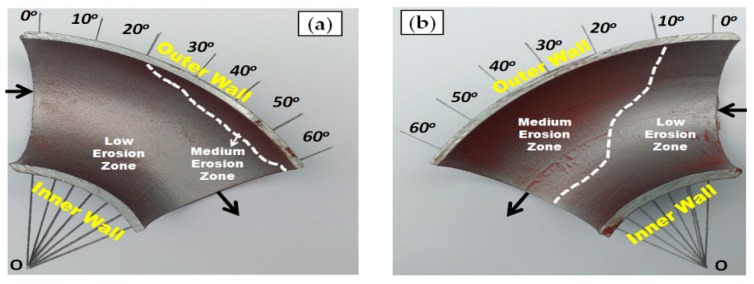
Paint erosion pattern for slug flow with Vs_L_ = 0.5 m/s, Vs_G_ = 2.5 m/s in a 60° horizontal–horizontal elbow section. (**a**) Bottom half; (**b**) upper half.

**Figure 7:**
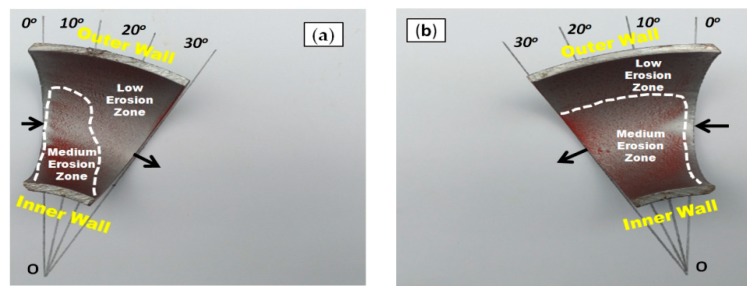
Paint erosion pattern for slug flow with Vs_L_ = 0.5 m/s, Vs_G_ = 2.5 m/s in a 30° horizontal–horizontal elbow section. (**a**) Bottom half; (**b**) upper half.

Figure 5, Figure 6 and Figure 7 illustrate the qualitative indication of the paint erosion distribution in the bottom half (BH) and upper half (UH) of the three different elbow specimens for slug flow, respectively. The MPM test conditions were slug flow pattern (Vs_G_ = 2.5 m/s and Vs_L_ = 0.5 m/s), fine sand particles with 2% (wt/wt) concentration, and flow time of 60 min for all cases (MPM tests without sand particles are reported in Figures S1–S3 (Supplementary Materials)). The extracted paint erosion distribution results in the bottom half (BH) and upper half (UH) sections of 90°, 60°, and 30° elbow configurations being compared to identify the highest erosion locations. A visual inspection indicates that an unsymmetrical erosion distribution is obtained for the horizontally oriented 90°, 60°, and 30° elbows, and less erosion is perceived in the bottom half (BH) section compared with the upper half (UH) elbow section for erosive slug flow conditions. The erosion distribution results with MPM techniques match closely with the research findings of other authors [20,22], and found that the top of the 90° elbow experiences more erosive damage in multiphase flow.

Figure 5 presents the extent of paint removal occurrence in the bottom half (BH) and the upper half (UH) sections of the 90° elbow when exposed to fine silica sand. The low, medium, and high eroded zones are clearly visible in the BH and UH sections after exposure to the slug flow regime. The high erosion zone extends between 60° and 90° in the upper half (UH) section (Figure 5b). The paint layer here is completely eroded, suggesting that severe particle impacts do occur compared with elsewhere on the 90° elbow pipe.

Figure 6 illustrates that the partially removed layers of paint on the outer wall were visible on the 60° elbow pipe surface, but are very minor compared with that of the 90° elbow pipe. In contrast, the medium erosion zone is observed in the inner wall of the 30° elbow (Figure 6) compared with the 60° elbow pipe with the medium erosion zone at the outer wall (Figure 7).

### 3.2. Surface Roughness

To quantitatively collate the surface roughness parameters of the 90°, 60°, and 30° elbows before and after exposure to erosive slug flow, a Sensofar 3D non-contact profiler was used to extract the surface roughness of the elbows’ section after 10 h of flow time, while before test surface roughness is taken from a Mitutoyo portable surface roughness tester.

**Figure 8:**
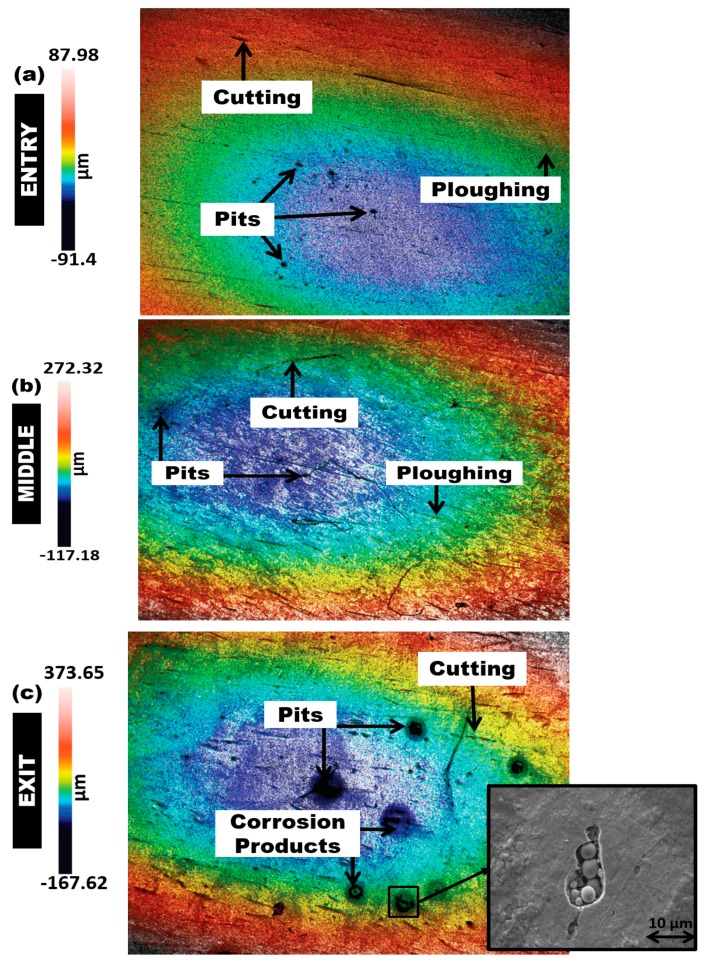
Confocal micrographs after erosion–corrosion: surface topographies of 90° carbon steel elbows. (**a**) Entry section; (**b**) middle section; (**c**) exit section.

**Figure 9:**
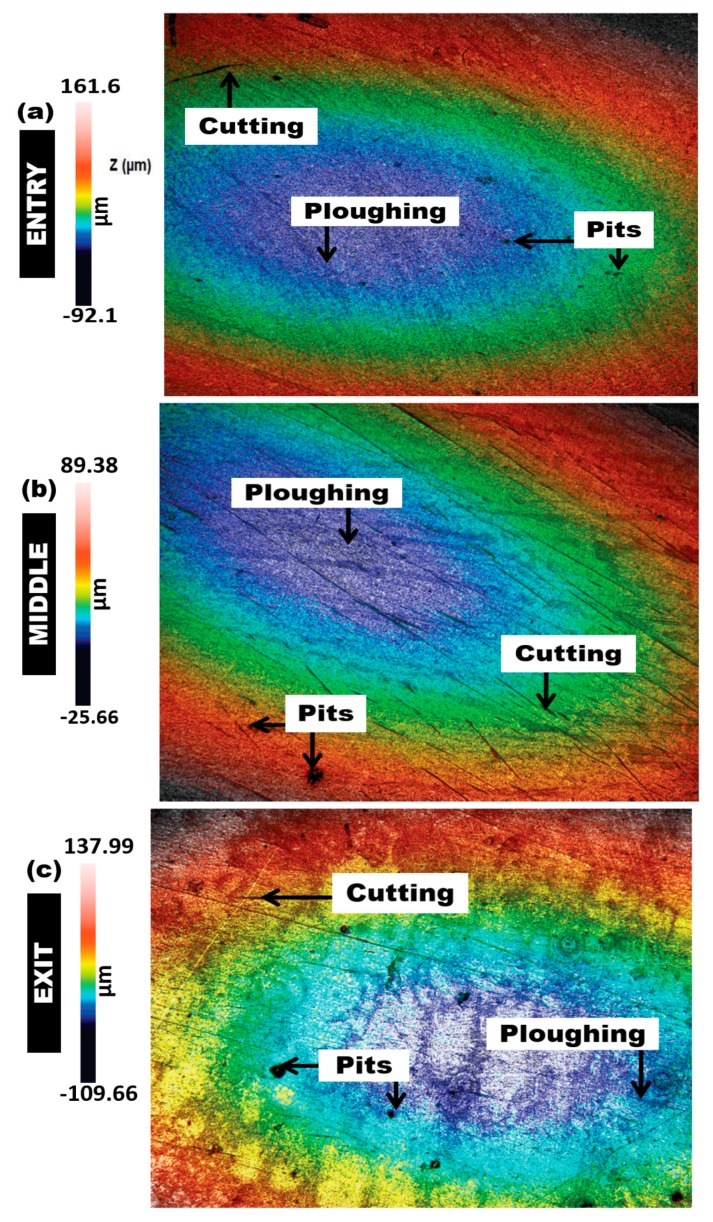
Confocal micrographs after erosion–corrosion: surface topographies of 60° carbon steel elbows. (**a**) Entry section; (**b**) middle section; (**c**) exit section.

**Figure 10:**
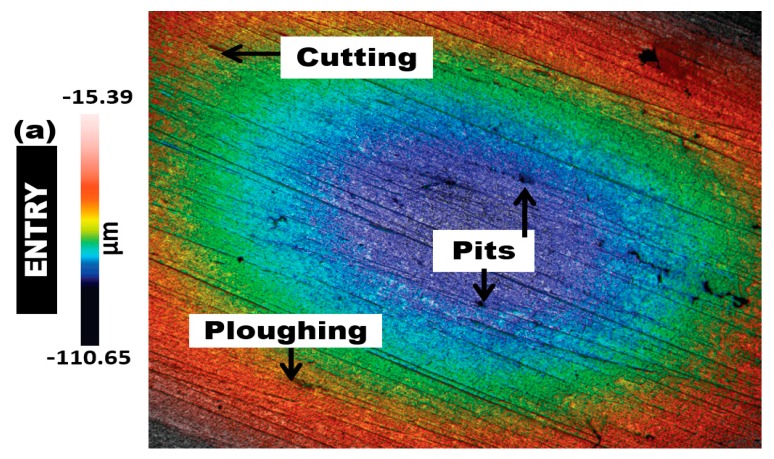

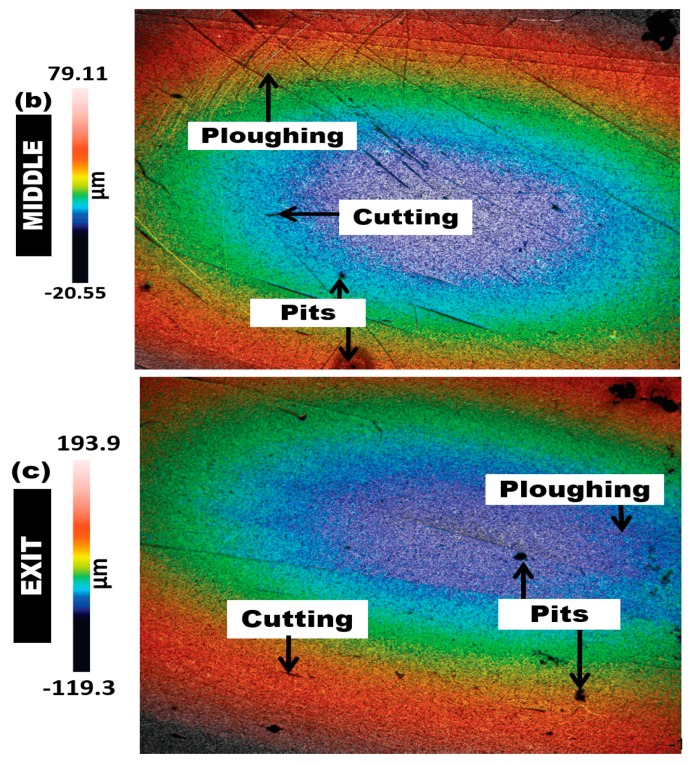
Confocal micrographs after erosion–corrosion: surface topographies of 30° carbon steel elbows. (**a**) Entry section; (**b**) middle section; (**c**) exit section.

Figure 8, Figure 9 and Figure 10 show the 3D surface topography scan taken from non-contact surface profiling of worn surfaces of the inlet, middle, and outlet sections of the elbows tested under erosive slug flow for 2% (wt/wt) sand concentration (surface profiles after the test are reported in Figures S4–S6 (Supplementary Materials)). It can be observed that the downstream section of the 90° and 60° eroded elbows are similar, while the 30° elbow shows more cutting wear with several erosion scars compared with the 90° and 60° elbow inlet section (Figure 8a, Figure 9a, and Figure 10a). In contrast, the magnitude of the erosion–corrosion degradation at the downstream adjacent to an outlet is severe for the 90° elbow configuration, which suggests that pitting action contributing to the overall erosion wear could be the dominant degradation mechanism, as shown in Figure 8c.

In the slug flow regime, micro-sized pits with cutting wear were spotted in the middle region of the 90° and 60° elbow configurations (Figure 8b and Figure 9b), while that of the entry region (Figure 8a and Figure 9a) appears to be less rough for the 90° and 60° elbow. In contrast, large pits with corrosion zones are observed adjacent to the outlet of the 90° and 60° elbows tested for the slug flow regime (Figure 8c and Figure 9c). This could be because the wide-angled elbow strongly influences particle–wall interactions and escalates the kinetic energy of particles and the consequent increase in material degradation. Generally, the difference in the wear mechanism across the elbows’ surface can be ascribed to the observation that the fine sand was transported with both air and water phases inside the pipe. In slug flow, sand mostly settles at the bottom of the elbow, and then the moving carrier phase aggregates the sand and induces acceleration. In addition, the sand concentration and impact velocity seem to be higher in the upper half section for slug flow [22,31], and resulted in a high turbulence zone owing to the high particle concentration and impact velocity in the upper half section of the elbow configurations.

The confocal micrographs of the wear surfaces of the 90°, 60°, and 30° elbows after performing the erosion–corrosion test, presented in Figure 8, Figure 9 and Figure 10, clearly indicate that the internal surfaces are dominated by corrosion attack and pitting erosion with cutting wear. This material degradation process seems to increase the extent of erosion–corrosion in the wide-angle elbow for slug flow conditions. However, the magnitude of the flow accelerated erosion–corrosion damage in the wide-angle elbow is more than those of the small-angle elbows under the same operating conditions. Under slug flow conditions, material removal in the 60° elbow also occurs by pitting, ploughing, and abrasive wear, but because of less penetration energy of particles, the magnitude of surface damage is disparaging, which is consistent with the internal surface topographies presented in this section. 

In the cutting mechanism (occurs at the exit surface of the 90° elbow), the erodent penetration energy is higher and the observed scars are shorter than the scar pattern obtained in the entry and middle sections. In addition, corrosion products with large pitting in the 90° and 60° exit section of the elbows (Figure 8c and Figure 9c) are symbols of plastic deformation owing to the repeated impact of the erodent. This implies that, in the wide-angle elbows’ configuration, the cumulative effect of cutting wear and impact wear enhances the erosion–corrosion damage.

On the basis of microscopic analysis, the identified dominant erosion–corrosion mechanisms are cutting and pitting, which occur at the surface of all specimens, regardless of angular configuration. However, larger erosion and/or corrosion pits only occur at the exit section, when the angular configuration is 90° and 60°, respectively. Corrosion attack and pitting dominates within an exit section of 90° and 60°, while the dominance of cutting wear in the case of the small-angle elbow is clearly visible in Figure 10.

**Figure 11:**
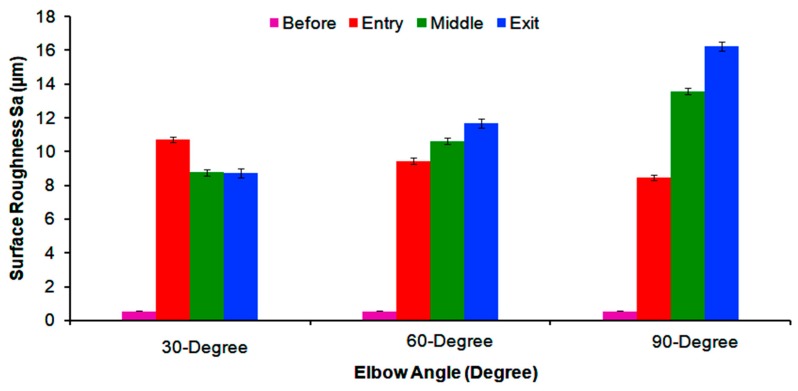
Surface roughness of the 30°, 60°, and 90° carbon steel horizontal elbow sections before and after erosion–corrosion.

Figure 11 shows the disparity in the surface roughness parameter, S_a_, for three different bend-angles before and after the test. The surface roughness increases as the flow approaches downstream and is consistently higher in the exit section of the 90° and 60° elbow after erosion–corrosion than that before erosion–corrosion, as well as those of the entry and middle sections, except at the 30° elbow, where the dominance of erosion scars on the entry section proliferates its surface roughness. This is because, with a small bend-angle, more particle wall collisions attribute to high turbulence at the upstream in the 30° elbow configurations. This observation supports previous speculation [16,32,33] that increased local turbulence/mass transfer resulted in a significant increase in surface roughness. In the 90° and 60° elbows, the exit region is dominated by cuttings wears and pitting actions with severe metal corrosion, which escalates the surface roughness. This implies that repeated impact by the fine sand adjacent to the outlet generates high penetration energy, which causes initiation and propagation of cutting wear and erosion–corrosion pitting. It was noticed that the surface roughness adjacent to the outlet after exposure to erosive slug flow increased from 8.64 ± 0.25 μm to 16.21 ± 0.46 μm as the elbow angle changed from 30° to 90°. In contrast, the surface roughness in the upstream section after the test decreased from 10.72 ± 0.34 μm to 8.46 ± 0.31 μm with an increase of the elbow angle from 30° to 90°, respectively.

**Figure 12:**
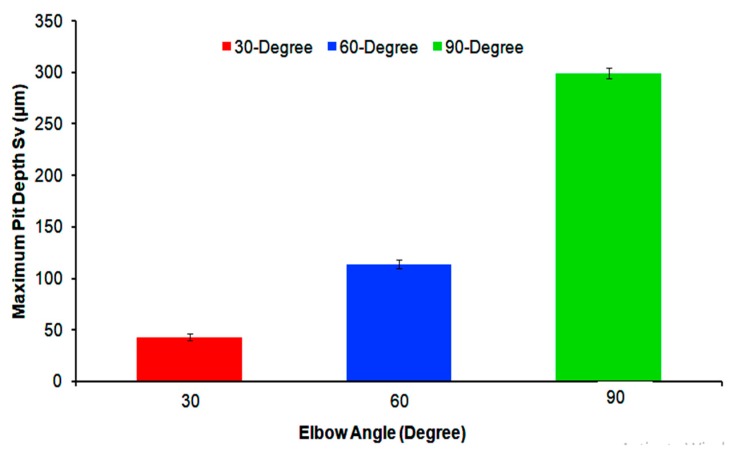
Maximum pit depth of 30°, 60°, and 90° carbon steel horizontal elbows outlet sections after erosion–corrosion.

It is clear from Figure 12 that, in the wide-angle elbow, the pit depth (S_v_) was increased, which indicates an increase in penetration energy. The maximum pit depth was observed at the exit section of the 90° elbow, and it was found to be 298.78 ± 9.71 µm. However, in the small-angle elbow case of erosion–corrosion, the fine particle impacts the surface with less penetration energy, and the maximum depth of 43.23 ± 5.48 µm was observed at the 30° elbows. At the 60° angle elbow, particles’ impacts at the exit section generate deeper pits, with a maximum depth of 113.89 ± 7.73 µm compared with the 30° elbow, as shown in Table 3.

**Figure 13:**
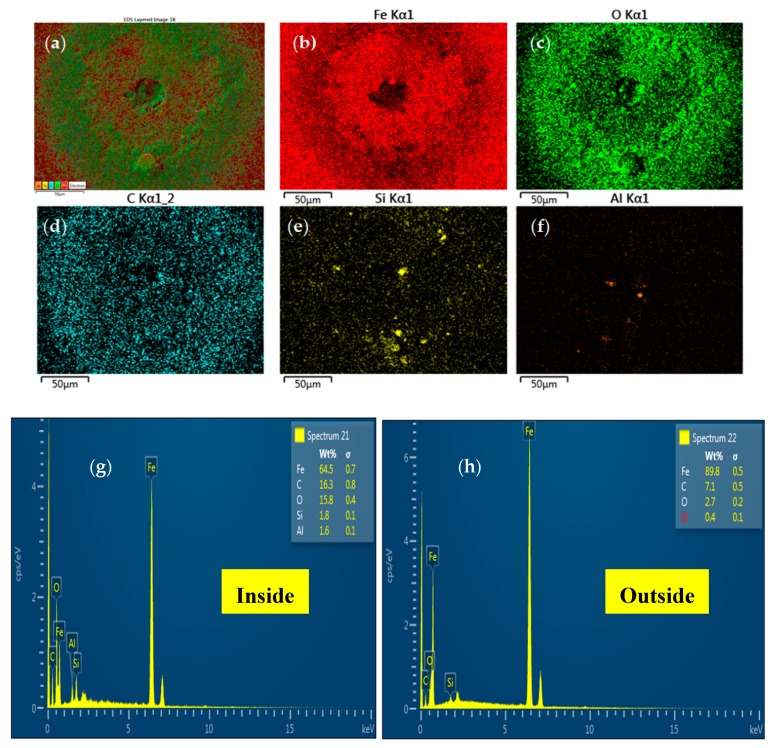
EDS analysis after erosion-corrosion. (**a**–**f**) Elemental map; (**g**,**h**) elemental phase spectra of the inside and outside of corrosion pits.

An EDS analysis of the worn surface was conducted to investigate the X-ray spectrum and distribution of the elemental phase after the erosion–corrosion test. Point scans were performed inside and outside the pit, and color-coded dot maps are shown for the individual phases in Figure 13.

The EDS spectrum reveals the dominance of iron atoms outside the pit on the surface (Figure 13h). However, the detected level of iron atoms reduced inside the pit (Figure 13g), while an escalation of the oxygen atoms was detected inside the pit. Therefore, it can be elucidated that the escalation in the level of the oxygen atom is a symbol that the pit contains iron oxide deposits and enhances localized corrosion inside the pit. It is important to highlight that the presence of Si (almost 0.4 wt.%) outside the pit and the increase in the level of Si (about 1.8 wt.%) inside the pit are evidence of severe particle impingement. 

### 3.3. Mass Loss

**Figure 14:**
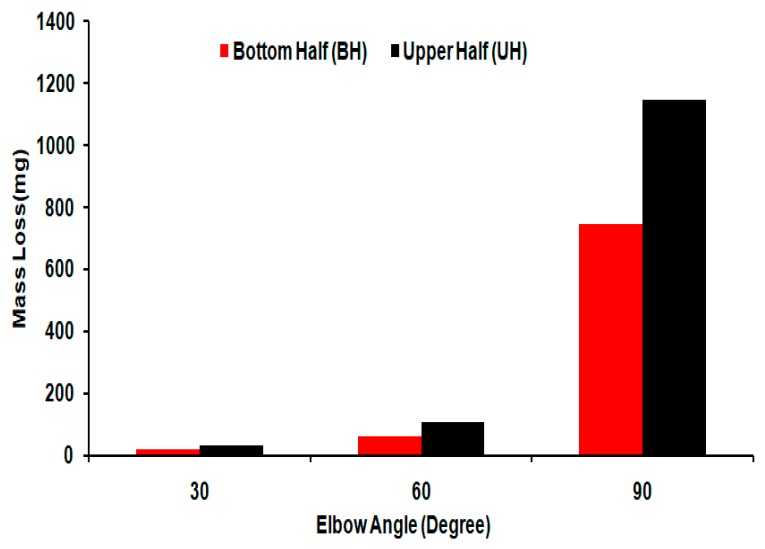
Mass loss in the carbon steel BH and UH elbow sections after exposure to slug flow for 10 h.

Figure 14 illustrates the representative mass loss for the 90°, 60°, and 30° elbows tested in slug flow containing 2 wt.%, particle concentrations, and fine silica sand. The mass loss of specimens was measured in the bottom half (BH) and the upper half (UH) elbow sections after being exposed to abrasive slug flow (Figure 14). As evident from Figure 14, the mass loss increased significantly with the change of elbow configuration to wide-angle, which may indicate that high erosive wear has taken place in the 90° elbow. Table 4 presents the influence of the elbow angle on the cumulative mass degradation rate after erosion–corrosion for the slug flow regime. In the present study, the mass-loss rate in the 90° angle elbow was 12 times higher than the mass loss rate in the 30° elbow, which implies severe particle–wall interaction in the wide-angle elbow, consequently increasing the erosion–corrosion rate of the wide-angle elbow (Table 4). This was because the 90° curvature angle resulted in higher turbulence as a result of the redirected and sliding particle, which may increase the erosive wear on the internal surface [33]. However, with the change in the elbow configuration from 30° to 60°, the increase in mass loss is minimal compared with the 90° elbow, which is evidence of less erosive wear for the slug flow conditions in the small-angle elbow configurations. It could also be because the decreasing elbow angle may decrease the direct particle to wall impact on the internal surface of the elbow [23,34]. It can be observed that mass loss is higher for UH than BH, and creates approximately 1.5 to 1.7 times greater metal loss in all cases. This implies more entrained sand in the gas core with high kinetic energy for slug flow, and resulted from sand impingement in the elbow upper half (UH) section, causing much more mass loss, which is consistent with the MPM pattern observed in Section 3.1. Similar experimental findings were reported by M. Parsi et al. [22].

## 4. Conclusions

A multiphase experimental setup was used to investigate the flow accelerated erosion–corrosion mechanism of 90°, 60°, and 30° carbon steel elbows for erosive slug flow containing 2% (wt/wt) sand concentrations. On the basis of the research findings, the following conclusions made:

- From mass loss rate measurements of the tested elbows, erosion–corrosion is observed to be 12 times higher in the wide-angle elbow than in the small-angle elbow for identical flow conditions.
- It was observed that the impact wear at the downstream of the 90° and 60° elbows precipitated in the formation of large erosion–corrosion pits and resulted in the highest erosive and corrosive damage adjacent to the elbow exit.
- For slug flow, the surface roughness on the elbow internal surface is greatly influenced by the elbow angular configuration. The mean surface roughness in the downstream adjacent to the outlet of the 90° and 60° elbow was higher than in the upstream regions, while at the 30° elbow, the surface roughness in the upstream region was higher than in the downstream region.
- In this work, the maximum erosive location was found in the upper half (UH) elbow section for all tested elbows, which experiences more erosion compared with the bottom half (BH) section.
- The extent of erosion–corrosion shows that the 90° elbow pipe is more prone to erosive damage than the 60° and 30° elbows in slug flow. Thus, to replace wide-angle elbows (e.g., 90°) with a small angle elbow (e.g., 30° and 60°) is recommended for suitable cases.

This work presents a contribution to the understanding of the erosion–corrosion mechanism of different elbow configurations in abrasive multiphase flow. A comprehensive understanding of the erosion–corrosion degradation process is essential in order to mitigate the induced damage due to erosion–corrosion in practice. Although the results obtained from the present study provided a good qualitative and quantitative comparison for multiphase flow, for a complete understanding of the erosion–corrosion mechanisms, further studies incorporating ultrasonic wall thickness loss measurements are necessary.

## Figures and Tables

**Table 1:** Composition of elbow material (wt.%).

Si	Cr	Cu	P	C	S	Ni	Mn	Fe
0.26	0.21	0.25	0.045	0.2	0.035	0.3	0.52	98.18

**Table 2:** Composition of silica sand (wt.%).

SiO_2_	Al_2_O_3_	Fe_2_O_3_	Na_2_O	MgO	CaO
98.08	1.17	0.28	0.03	0.22	0.22

**Table 3:** Surface parameters of elbows after erosion–corrosion.

Surface Parameters (µm)	90-Degree		60-Degree		30-Degree	
(µ ± σ)	S_a_	S_v_	S_a_	S_v_	S_a_	S_v_
Entry	8.46 ± 0.31	22.99 ± 0.42	9.45 ± 0.39	32.52 ± 1.12	10.72 ± 0.34	38.01 ± 4.63
Middle	13.56 ± 0.16	230.78 ± 2.41	10.62 ± 0.14	26.55 ± 1.81	8.76 ± 0.14	24.37 ± 3.89
Exit	16.21 ± 0.46	298.94 ± 9.17	11.63 ± 0.28	113.89 ± 7.73	8.64 ± 0.25	43.23 ± 5.48

**Table 4:** Mass loss rates of tested carbon steel elbows for slug flow.

Test Elbow	V_SG_ (m/s)	V_SL_ (m/s)	Flow Time (h)	Particle Size (µm)	Particle Concentration (wt.%)	Mass Loss Rate (kg/m^2^·s)
30°	2.5	0.5	10	50 ± 2	2	2.31 × 10^−7^
60°	2.5	0.5	10	50 ± 2	2	3.78 × 10^−7^
90°	2.5	0.5	10	50 ± 2	2	2.77 × 10^−6^

## Supplementary Materials

The following are available online at https://www.mdpi.com/1996-1944/12/23/3898/s1, Figure S1: Paint erosion pattern for slug flow without sand with Vs_L_ = 0.5 m/s, Vs_G_ = 2.5 m/s in a 90° horizontal–horizontal elbow section. (a) Bottom half; (b) upper half, Figure S2: Paint erosion pattern for slug flow without sand with Vs_L_ = 0.5 m/s, Vs_G_ = 2.5 m/s in a 60° horizontal–horizontal elbow section. (a) Bottom half; (b) upper half, Figure S3: Paint erosion pattern for slug flow without sand with Vs_L_ = 0.5 m/s, Vs_G_ = 2.5 m/s in a 30° horizontal–horizontal elbow section. (a) Bottom half; (b) upper half, Figure S4: Surface topographies and 2D profiles of 90° carbon steel elbows after erosion–corrosion. (a) Entry section; (b) middle section; (c) exit section, Figure S5: Topographies and 2D profiles of 60° carbon steel elbows after erosion–corrosion. (a) Entry section; (b) middle section; (c) exit section, Figure S6: Topographies and 2D profiles of 30° carbon steel elbows after erosion–corrosion. (a) Entry section; (b) middle section; (c) exit section.
