# Supplementary material for: Erosion–Corrosion of 30°, 60°, and 90° Carbon Steel Elbows in a Multiphase Flow Containing Sand Particles
Source: Materials (Basel). 2019 Nov 26;12(23):3898. doi: 10.3390/ma12233898 (PMC6926515; doi:10.3390/ma12233898)
Supplement: Supplementary file 1 [file materials-12-03898-s001.pdf]

materials

Supplementary Materials

Erosion–Corrosion of 30°, 60°, and 90° Carbon Steel Elbows in a Multiphase Flow Containing Sand Particles

Rehan Khan [1,*], Hamdan H. Ya [1,*], William Pao [1], and Armaghan Khan [2]

[1] Mechanical Engineering Department, Universiti Teknologi PETRONAS, Perak and Malaysia; william.pao@utp.edu.my

[2] Department of Mechanical Engineering, McGill University, Macdonald Engineering Building, 817 Sherbrooke Street West Montreal, Canada; armaghan.khan@mail.mcgill.ca

* Correspondence: Muhammad_15001294@utp.edu.my (R.K.); hamdan.ya@utp.edu.my (H.H.Y.); Tel.: +60136980670 (R.K.); +60194607706 (H.H.Y.)

Received: 5 September 2019; Accepted: 17 October 2019; Published: date

1. Multilayer Paint Modeling (MPM) for Slug Flow without Abrasive Particles

The paint removed pattern was analyzed without sand particles for identical test conditions in order to understand the influence of flow conditions.

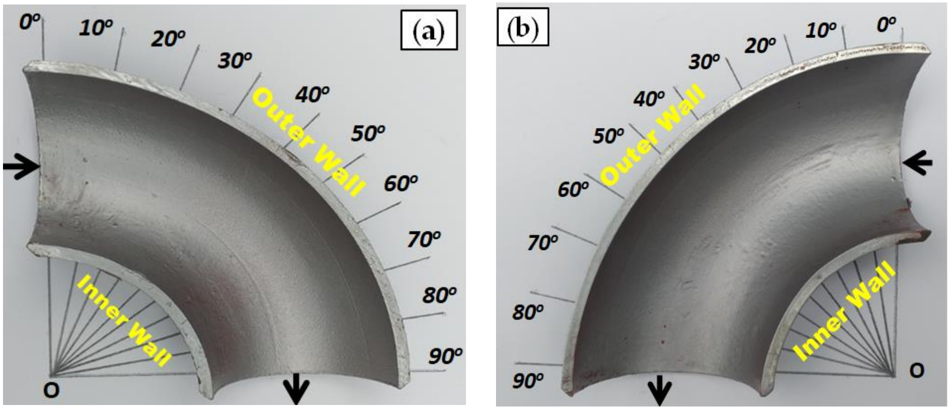

(a) (b)

Figure S1. Paint erosion pattern for slug flow without sand with V_{SL} = 0.5 m/s, V_{SG} = 2.5 m/s in a 90° horizontal–horizontal elbow section. (**a**) Bottom half; (**b**) upper half.

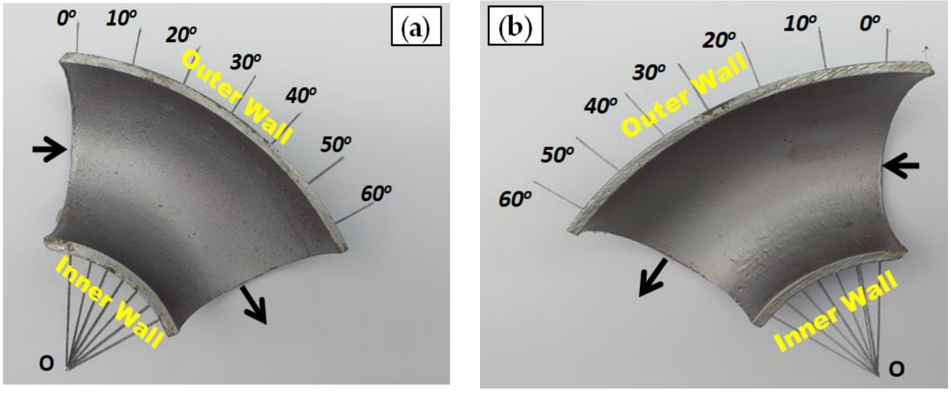

(a) (b)

Figure S2. Paint erosion pattern for slug flow without sand with V_{SL} = 0.5 m/s, V_{SG} = 2.5 m/s in a 60° horizontal–horizontal elbow section. (**a**) Bottom half; (**b**) upper half.

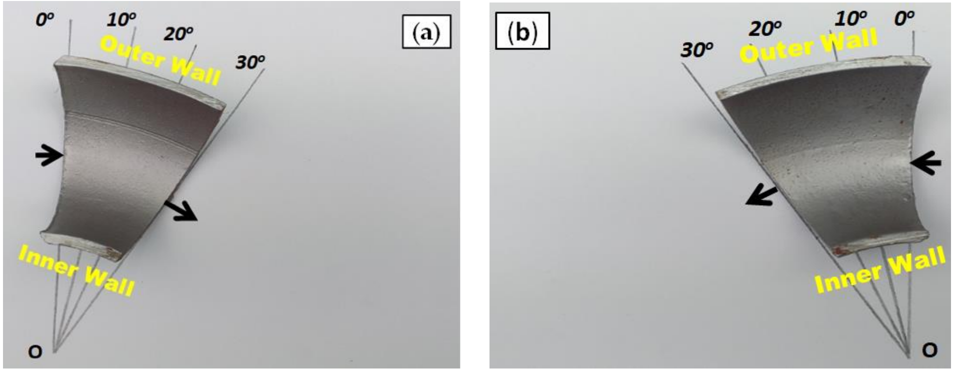

(**a**) (**b**)

Figure S3. Paint erosion pattern for slug flow without sand with V_{SL} = 0.5 m/s, V_{SG} = 2.5 m/s in a 30° horizontal–horizontal elbow section. (**a**) Bottom half; (**b**) upper half.

2. 3D Surface Topographies and 2D Profiles after Test

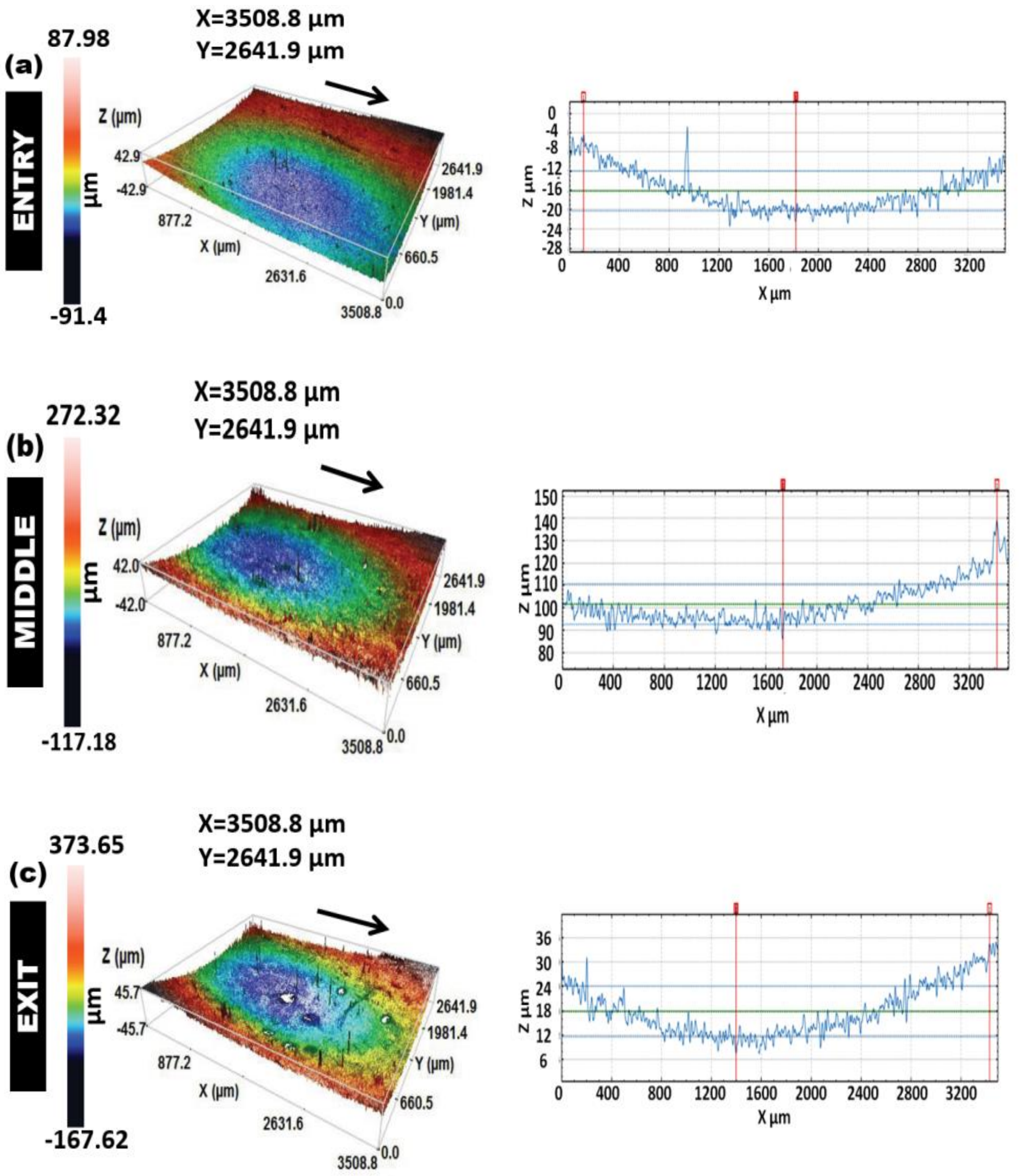

Figure S4. Surface topographies and 2D profiles of 90° carbon steel elbows after erosion–corrosion. (**a**) Entry section; (**b**) middle section; (**c**) exit section.

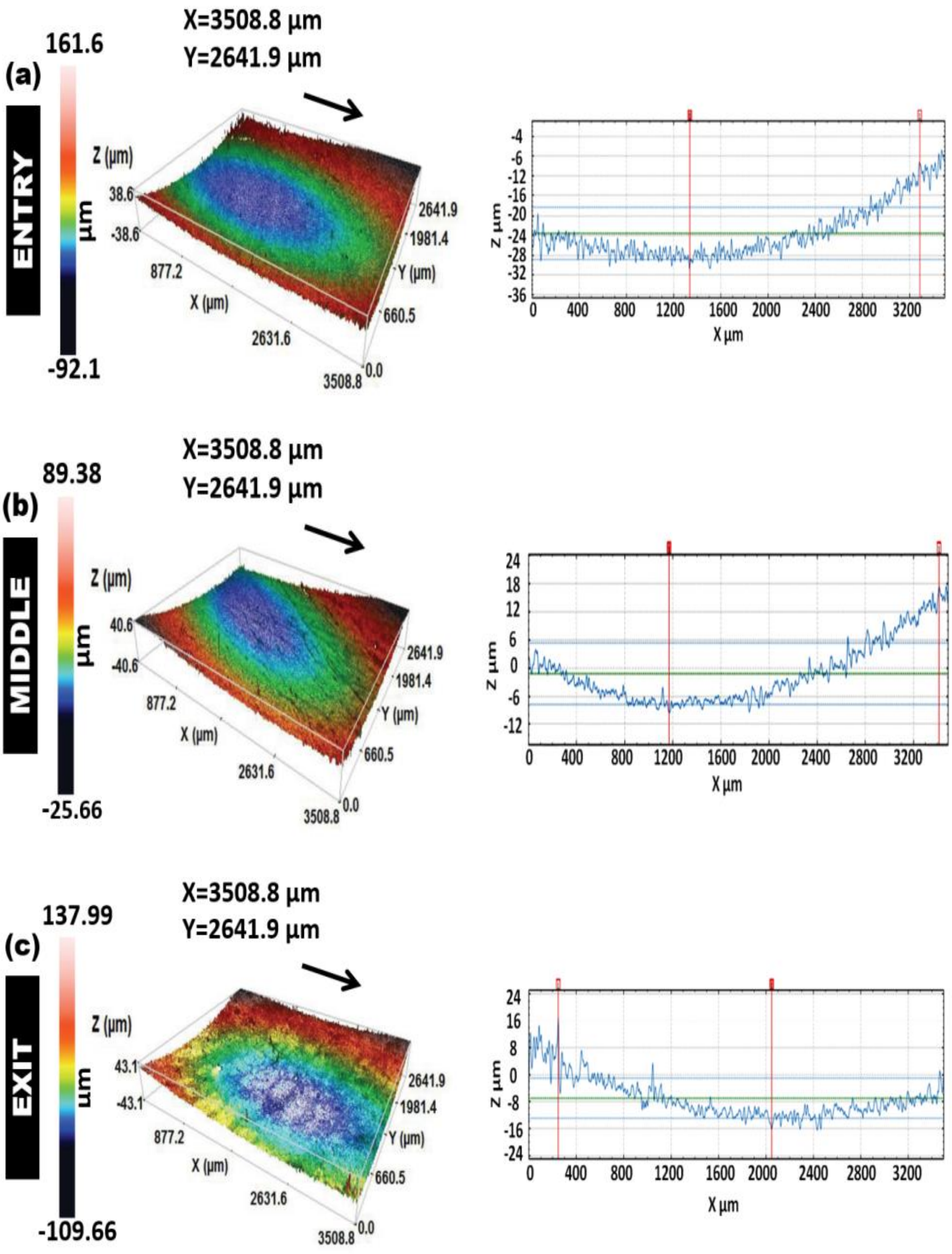

Figure S5. Topographies and 2D profiles of 60° carbon steel elbows after erosion–corrosion. (**a**) Entry section; (**b**) middle section; (**c**) exit section.

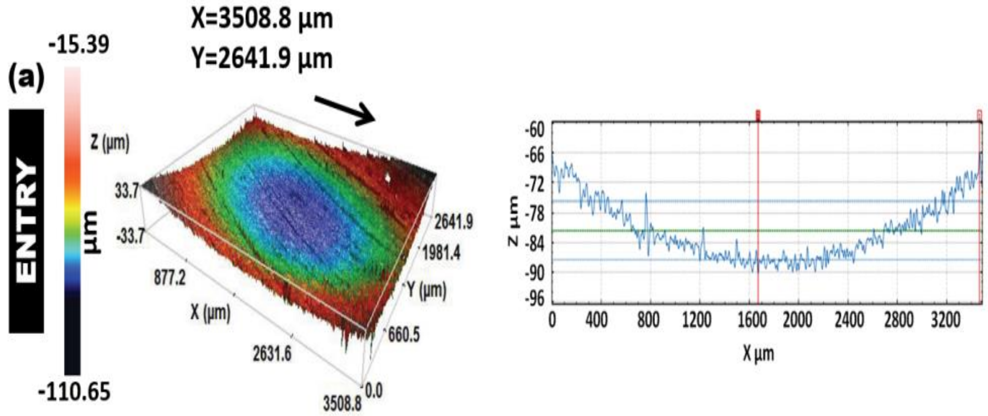

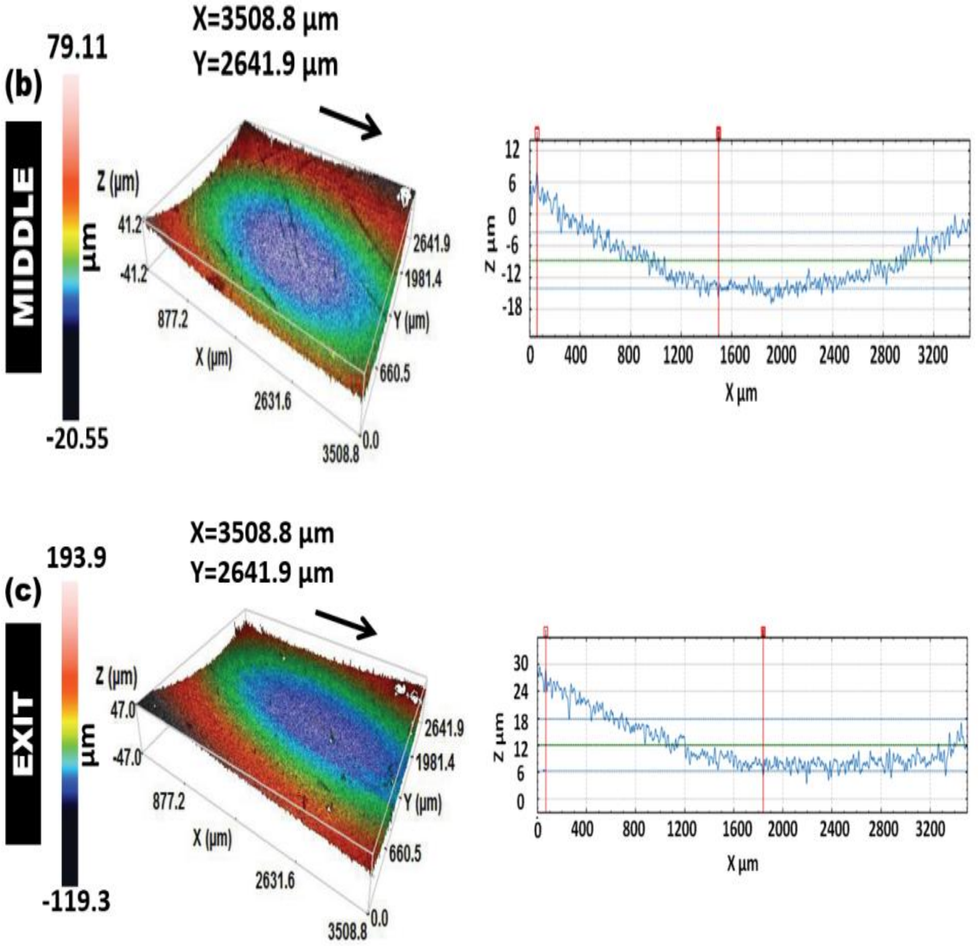

Figure S6. Topographies and 2D profiles of 30° carbon steel elbows after erosion–corrosion. (**a**) Entry section; (**b**) middle section; (**c**) exit section.
